# Disodium tetra­aqua­bis(sulfato)iron(II)

**DOI:** 10.1107/S1600536808000214

**Published:** 2008-01-09

**Authors:** Martin Hudák, Jesús García Díaz, Jozef Kožíšek

**Affiliations:** a Institute of Physical Chemistry and Chemical Physics, Slovak University of Technology, Radlinského 9, 81237 Bratislava, Slovak Republic; b Materials Degree, Institute Technologic of Morelia, Michoacán, Mexico

## Abstract

In the title compound, [FeNa_2_(SO_4_)_2_(H_2_O)_4_], the Fe^2+^ cation is situated on a centre of inversion and is hexa­coordinated by four O atoms from water mol­ecules and two O atoms from two sulfate anions in an octa­hedral geometry. The coordination environment of Na^+^ comprises six O atoms in a more distorted octa­hedral arrangement, with Na—O distances between 2.368 (1) and 2.612 (1) Å. The structure contains an extensive three-dimensional network of O—H⋯O hydrogen bonds.

## Related literature

For related structures, see: Held (2003 ▶); Barton *et al.* (2002 ▶).

## Experimental

### 

#### Crystal data

[FeNa_2_(SO_4_)_2_(H_2_O)_4_]
*M* *_r_* = 366.01
Monoclinic, 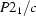

*a* = 5.551 (1) Å
*b* = 8.252 (1) Å
*c* = 11.162 (1) Å
β = 100.20 (1)°
*V* = 503.1 (1) Å^3^
*Z* = 2
Mo *K*α radiation
μ = 2.06 mm^−1^
*T* = 301 (2) K
0.55 × 0.44 × 0.17 mm

#### Data collection

Oxford Diffraction Gemini R CCD diffractometer
Absorption correction: analytical [*CrysAlis RED* (Oxford Diffraction, 2007 ▶), based on Clark & Reid (1995 ▶)] *T*_min_ = 0.398, *T*_max_ = 0.720
19525 measured reflections
1343 independent reflections
1288 reflections with *I* > 2σ(*I*)
*R*_int_ = 0.022

#### Refinement

*R*[*F* ^2^ > 2σ(*F* ^2^)] = 0.018
*wR*(*F* ^2^) = 0.047
*S* = 1.07
1343 reflections
96 parameters
All H-atom parameters refined
Δρ_max_ = 0.31 e Å^−3^
Δρ_min_ = −0.40 e Å^−3^

### 

Data collection: *CrysAlis CCD* (Oxford Diffraction, 2007 ▶); cell refinement: *CrysAlis RED* (Oxford Diffraction, 2007 ▶); data reduction: *CrysAlis RED*; program(s) used to solve structure: *SHELXS97* (Sheldrick, 2008 ▶); program(s) used to refine structure: *SHELXL97* (Sheldrick, 2008 ▶); molecular graphics: *DIAMOND* (Brandenburg, 1998 ▶); software used to prepare material for publication: *enCIFer* (Allen *et al.*, 2004 ▶).

## Supplementary Material

Crystal structure: contains datablocks global, I. DOI: 10.1107/S1600536808000214/bi2271sup1.cif

Structure factors: contains datablocks I. DOI: 10.1107/S1600536808000214/bi2271Isup2.hkl

Additional supplementary materials:  crystallographic information; 3D view; checkCIF report

## Figures and Tables

**Table 1:** Hydrogen-bond geometry (Å, °)

*D*—H⋯*A*	*D*—H	H⋯*A*	*D*⋯*A*	*D*—H⋯*A*
O8—H8*A*⋯O5^i^	0.78 (3)	1.97 (3)	2.7513 (15)	172 (2)
O8—H8*B*⋯O6^ii^	0.73 (3)	2.00 (3)	2.7116 (15)	165 (2)
O9—H9*A*⋯O6^iii^	0.74 (3)	2.16 (3)	2.8741 (14)	162 (2)
O9—H9*B*⋯O5^i^	0.70 (3)	2.32 (3)	2.9379 (15)	150 (3)

Symmetry codes: (i) 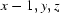; (ii) 
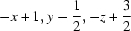
; (iii) 
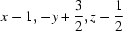
.

**Fig. 1.**
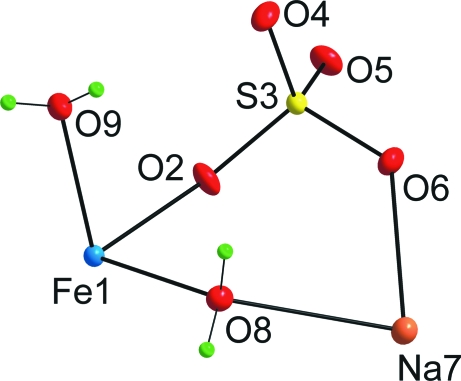
The asymmetric unit of the title compound with displacement ellipsoids shown at the 30% probability level for non-H atoms.

**Fig. 2.**
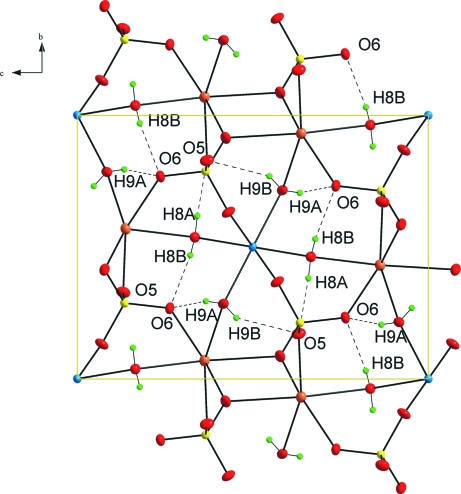
Packing diagram viewed along the a axis. Hydrogen-bond interactions are indicated by dashed lines.

## supplementary crystallographic information

### Comment

In order to find convenient crystals as model compounds for investigation of the electronic structure of Fe^II^ complexes, the title compound was prepared. The unit cell contains two Fe^II^ cations situated on centres of inversion, four Na^+^ cations, four SO_4_^2-^ anions and eight water molecules. The structure contains a three-dimensional network of O—H···O hydrogen bonds.

In the title compound (Fig. 1), the Fe1—O8 [2.115 (1) Å] and Fe1—O9 [2.170 (1) Å] bond lengths are in reasonable agreement with a similar compounds in the Cambridge Structural Database (CSD; Version 5.27, 2006 release). For example, in ethylenediammonium tetra-aqua-bis(sulfato-O)-iron(II) (Held, 2003; CSD refcode WABHIR), the Fe—O(water) distances are 2.111 and 2.143 Å, respectively. The Fe1—O2 distance to the sulfate anion [2.095 (1) Å] compares to 2.126 Å in WABHIR. In the crystal structure of pentaaqua-(3-(diphenylphosphino)phenylsulfonato)-iron(II) (diphenyl(3-sulfonatophenyl)phosphine) (Barton *et al.*, 2002; CSD refcode XOHHOR), there are two symmetrically independent units with corresponding distances for Fe—O(water) in the range 2.085 to 2.125 Å and for Fe—O of the sulfonato anion between 2.10 and 2.126 Å.

### Experimental

A solution of 1.0 mmol of Fe(SO_4_)_2_ in 2 ml water with one drop of diluted H_2_SO_4_(aq) and a piece of Fe metal was mixed with a solution of 2.0 mmol NaNO_2_NCN in 10 ml water and a solution of 2.0 mmol of imidazole in 10 ml me thanol. From this system, pale yellow crystals were isolated after a few days standing.

### Refinement

H atoms were located in difference Fourier maps and refined freely with isotropic displacement parameters.

### Crystal data

**Table:**

[FeNa_2_(SO_4_)_2_(H_2_O)_4_]	*F*_000_ = 368
*M**_r_* = 366.01	*D*_x_ = 2.416 Mg m^−^^3^
Monoclinic, *P*2_1_/*c*	Mo *K*α radiation λ = 0.71073 Å
Hall symbol: -P 2ybc	Cell parameters from 17533 reflections
*a* = 5.551 (1) Å	θ = 3.0–31.4º
*b* = 8.252 (1) Å	µ = 2.06 mm^−^^1^
*c* = 11.162 (1) Å	*T* = 301 (2) K
β = 100.20 (1)º	Block, pale yellow
*V* = 503.1 (1) Å^3^	0.55 × 0.44 × 0.17 mm
*Z* = 2	

### Data collection

**Table:**

Oxford Diffraction Gemini R CCD diffractometer	1343 independent reflections
Radiation source: fine-focus sealed tube	1288 reflections with *I* > 2σ(*I*)
Monochromator: graphite	*R*_int_ = 0.022
*T* = 301(2) K	θ_max_ = 29.5º
ω and φ scans	θ_min_ = 6.6º
Absorption correction: analytical[CrysAlis RED (Oxford Diffraction, 2007), based on Clark & Reid (1995)]	*h* = −7→7
*T*_min_ = 0.398, *T*_max_ = 0.720	*k* = −11→11
19525 measured reflections	*l* = −15→15

### Refinement

**Table:**

Refinement on *F*^2^	Hydrogen site location: inferred from neighbouring sites
Least-squares matrix: full	All H-atom parameters refined
*R*[*F*^2^ > 2σ(*F*^2^)] = 0.018	*w* = 1/[σ^2^(*F*_o_^2^) + (0.0245*P*)^2^ + 0.3061*P*] where *P* = (*F*_o_^2^ + 2*F*_c_^2^)/3
*wR*(*F*^2^) = 0.047	(Δ/σ)_max_ = 0.001
*S* = 1.08	Δρ_max_ = 0.31 e Å^−^^3^
1343 reflections	Δρ_min_ = −0.40 e Å^−^^3^
96 parameters	Extinction correction: SHELXL97 (Sheldrick, 1997), Fc^*^=kFc[1+0.001xFc^2^λ^3^/sin(2θ)]^-1/4^
Primary atom site location: structure-invariant direct methods	Extinction coefficient: 0.062 (3)
Secondary atom site location: difference Fourier map	

### Special details

**Table:**

Geometry. All e.s.d.'s (except the e.s.d. in the dihedral angle between two l.s. planes) are estimated using the full covariance matrix. The cell e.s.d.'s are taken into account individually in the estimation of e.s.d.'s in distances, angles and torsion angles; correlations between e.s.d.'s in cell parameters are only used when they are defined by crystal symmetry. An approximate (isotropic) treatment of cell e.s.d.'s is used for estimating e.s.d.'s involving l.s. planes.
Refinement. Refinement of *F*^2^ against ALL reflections. The weighted *R*-factor *wR* and goodness of fit *S* are based on *F*^2^, conventional *R*-factors *R* are based on *F*, with *F* set to zero for negative *F*^2^. The threshold expression of *F*^2^ > σ(*F*^2^) is used only for calculating *R*-factors(gt) *etc*. and is not relevant to the choice of reflections for refinement. *R*-factors based on *F*^2^ are statistically about twice as large as those based on *F*, and *R*- factors based on ALL data will be even larger.

### Fractional atomic coordinates and isotropic or equivalent isotropic displacement parameters (Å^2^)

**Table:**

	*x*	*y*	*z*	*U*_iso_*/*U*_eq_	
O2	0.81620 (17)	0.63212 (12)	0.56957 (10)	0.0228 (2)	
O4	0.70650 (19)	0.91424 (13)	0.57758 (9)	0.0234 (2)	
O5	1.12799 (17)	0.82849 (12)	0.63063 (10)	0.0234 (2)	
O6	0.84525 (19)	0.76778 (12)	0.76330 (9)	0.0224 (2)	
O8	0.37101 (18)	0.53699 (14)	0.66515 (9)	0.01827 (19)	
H8A	0.294 (4)	0.617 (3)	0.660 (2)	0.039 (6)*	
H8B	0.290 (4)	0.472 (3)	0.679 (2)	0.031 (6)*	
O9	0.31917 (19)	0.71528 (13)	0.41612 (10)	0.0201 (2)	
H9A	0.211 (5)	0.707 (3)	0.368 (2)	0.039 (6)*	
H9B	0.272 (5)	0.771 (3)	0.453 (2)	0.040 (7)*	
S3	0.86989 (5)	0.78740 (3)	0.63455 (3)	0.01276 (9)	
Na7	0.62761 (10)	0.56900 (7)	0.86236 (5)	0.02189 (13)	
Fe1	0.5000	0.5000	0.5000	0.01297 (9)	

### Atomic displacement parameters (Å^2^)

**Table:**

	*U*^11^	*U*^22^	*U*^33^	*U*^12^	*U*^13^	*U*^23^
O2	0.0175 (4)	0.0203 (5)	0.0302 (5)	−0.0039 (3)	0.0031 (4)	−0.0113 (4)
O4	0.0263 (5)	0.0248 (5)	0.0183 (4)	0.0113 (4)	0.0017 (4)	0.0034 (4)
O5	0.0148 (4)	0.0218 (5)	0.0342 (5)	−0.0049 (4)	0.0058 (4)	−0.0040 (4)
O6	0.0292 (5)	0.0243 (5)	0.0142 (4)	0.0024 (4)	0.0054 (4)	0.0024 (4)
O8	0.0190 (4)	0.0188 (5)	0.0182 (4)	0.0011 (4)	0.0068 (3)	0.0005 (4)
O9	0.0203 (5)	0.0178 (5)	0.0198 (5)	0.0025 (4)	−0.0030 (4)	−0.0015 (4)
S3	0.01181 (14)	0.01334 (15)	0.01272 (15)	0.00036 (9)	0.00107 (10)	−0.00092 (10)
Na7	0.0242 (3)	0.0222 (3)	0.0185 (3)	−0.0010 (2)	0.0018 (2)	0.0011 (2)
Fe1	0.01386 (13)	0.01298 (13)	0.01206 (13)	−0.00055 (8)	0.00225 (8)	−0.00057 (8)

### Geometric parameters (Å, °)

**Table:**

O2—S3	1.4765 (10)	O9—Na7^i^	2.6122 (12)
O2—Fe1	2.0951 (9)	O9—H9A	0.74 (3)
O4—S3	1.4553 (10)	O9—H9B	0.70 (3)
O4—Na7^i^	2.3683 (11)	S3—Na7^i^	3.3160 (6)
O4—Na7^ii^	2.4413 (11)	Na7—O4^iv^	2.3684 (11)
O5—S3	1.4805 (9)	Na7—O5^v^	2.3974 (11)
O5—Na7^iii^	2.3975 (11)	Na7—O4^vi^	2.4413 (11)
O6—S3	1.4764 (10)	Na7—O9^iv^	2.6123 (12)
O6—Na7	2.4178 (11)	Na7—S3^iv^	3.3160 (6)
O8—Fe1	2.1146 (9)	Na7—Na7^vii^	3.7798 (11)
O8—Na7	2.4153 (11)	Na7—H8B	2.64 (2)
O8—H8A	0.78 (3)	Fe1—O2^viii^	2.0951 (9)
O8—H8B	0.73 (3)	Fe1—O8^viii^	2.1146 (9)
O9—Fe1	2.1695 (10)	Fe1—O9^viii^	2.1695 (10)
			
S3—O2—Fe1	135.77 (6)	O8—Na7—O9^iv^	88.81 (4)
S3—O4—Na7^i^	118.30 (6)	O6—Na7—O9^iv^	92.51 (4)
S3—O4—Na7^ii^	134.52 (6)	O4^vi^—Na7—O9^iv^	74.50 (4)
Na7^i^—O4—Na7^ii^	103.59 (4)	O4^iv^—Na7—S3^iv^	22.73 (2)
S3—O5—Na7^iii^	137.19 (6)	O5^v^—Na7—S3^iv^	97.50 (3)
S3—O6—Na7	131.26 (6)	O8—Na7—S3^iv^	162.20 (3)
Fe1—O8—Na7	125.04 (5)	O6—Na7—S3^iv^	91.27 (3)
Fe1—O8—H8A	108.5 (17)	O4^vi^—Na7—S3^iv^	98.04 (3)
Na7—O8—H8A	102.0 (17)	O9^iv^—Na7—S3^iv^	73.86 (3)
Fe1—O8—H8B	113.3 (17)	O4^iv^—Na7—Na7^vii^	38.89 (3)
Na7—O8—H8B	99.8 (17)	O5^v^—Na7—Na7^vii^	90.89 (3)
H8A—O8—H8B	106 (2)	O8—Na7—Na7^vii^	117.55 (3)
Fe1—O9—Na7^i^	112.65 (4)	O6—Na7—Na7^vii^	149.97 (4)
Fe1—O9—H9A	120.0 (19)	O4^vi^—Na7—Na7^vii^	37.52 (2)
Na7^i^—O9—H9A	111.8 (19)	O9^iv^—Na7—Na7^vii^	70.32 (3)
Fe1—O9—H9B	118 (2)	S3^iv^—Na7—Na7^vii^	60.819 (15)
Na7^i^—O9—H9B	91 (2)	O4^iv^—Na7—H8B	141.4 (5)
H9A—O9—H9B	99 (3)	O5^v^—Na7—H8B	95.4 (5)
O4—S3—O6	110.26 (6)	O8—Na7—H8B	15.9 (5)
O4—S3—O2	110.70 (6)	O6—Na7—H8B	101.2 (5)
O6—S3—O2	109.73 (6)	O4^vi^—Na7—H8B	65.5 (5)
O4—S3—O5	110.72 (6)	O9^iv^—Na7—H8B	88.8 (5)
O6—S3—O5	108.11 (6)	S3^iv^—Na7—H8B	159.2 (5)
O2—S3—O5	107.24 (6)	Na7^vii^—Na7—H8B	102.8 (5)
O6—S3—Na7^i^	146.99 (4)	O2^viii^—Fe1—O2	180.0
O2—S3—Na7^i^	81.15 (5)	O2^viii^—Fe1—O8	90.43 (4)
O5—S3—Na7^i^	97.39 (5)	O2—Fe1—O8	89.57 (4)
O4^iv^—Na7—O5^v^	90.76 (4)	O2^viii^—Fe1—O8^viii^	89.57 (4)
O4^iv^—Na7—O8	154.70 (4)	O2—Fe1—O8^viii^	90.43 (4)
O5^v^—Na7—O8	100.26 (4)	O8—Fe1—O8^viii^	180.0
O4^iv^—Na7—O6	114.00 (4)	O2^viii^—Fe1—O9^viii^	91.55 (4)
O5^v^—Na7—O6	104.53 (4)	O2—Fe1—O9^viii^	88.45 (4)
O8—Na7—O6	85.36 (4)	O8—Fe1—O9^viii^	86.80 (4)
O4^iv^—Na7—O4^vi^	76.41 (4)	O8^viii^—Fe1—O9^viii^	93.20 (4)
O5^v^—Na7—O4^vi^	90.63 (4)	O2^viii^—Fe1—O9	88.45 (4)
O8—Na7—O4^vi^	80.73 (4)	O2—Fe1—O9	91.55 (4)
O6—Na7—O4^vi^	161.06 (4)	O8—Fe1—O9	93.20 (4)
O4^iv^—Na7—O9^iv^	74.82 (4)	O8^viii^—Fe1—O9	86.80 (4)
O5^v^—Na7—O9^iv^	161.21 (4)	O9^viii^—Fe1—O9	179.999 (1)
			
Na7^i^—O4—S3—O6	−164.83 (6)	Fe1—O8—Na7—O9^iv^	142.28 (6)
Na7^ii^—O4—S3—O6	−10.15 (11)	Fe1—O8—Na7—S3^iv^	129.32 (9)
Na7^i^—O4—S3—O2	−43.22 (8)	Fe1—O8—Na7—Na7^vii^	−150.72 (5)
Na7^ii^—O4—S3—O2	111.45 (9)	S3—O6—Na7—O4^iv^	172.17 (7)
Na7^i^—O4—S3—O5	75.58 (8)	S3—O6—Na7—O5^v^	74.68 (8)
Na7^ii^—O4—S3—O5	−129.75 (9)	S3—O6—Na7—O8	−24.74 (8)
Na7^ii^—O4—S3—Na7^i^	154.67 (13)	S3—O6—Na7—O4^vi^	−67.51 (16)
Na7—O6—S3—O4	101.13 (8)	S3—O6—Na7—O9^iv^	−113.34 (8)
Na7—O6—S3—O2	−21.05 (9)	S3—O6—Na7—S3^iv^	172.76 (7)
Na7—O6—S3—O5	−137.70 (7)	S3—O6—Na7—Na7^vii^	−166.63 (6)
Na7—O6—S3—Na7^i^	83.55 (10)	S3—O2—Fe1—O8	−46.73 (10)
Fe1—O2—S3—O4	−45.49 (11)	S3—O2—Fe1—O8^viii^	133.27 (10)
Fe1—O2—S3—O6	76.42 (10)	S3—O2—Fe1—O9^viii^	−133.54 (10)
Fe1—O2—S3—O5	−166.38 (9)	S3—O2—Fe1—O9	46.46 (10)
Fe1—O2—S3—Na7^i^	−71.33 (9)	Na7—O8—Fe1—O2^viii^	150.22 (6)
Na7^iii^—O5—S3—O4	32.62 (11)	Na7—O8—Fe1—O2	−29.78 (6)
Na7^iii^—O5—S3—O6	−88.26 (10)	Na7—O8—Fe1—O9^viii^	58.69 (6)
Na7^iii^—O5—S3—O2	153.49 (9)	Na7—O8—Fe1—O9	−121.31 (6)
Na7^iii^—O5—S3—Na7^i^	70.50 (9)	Na7^i^—O9—Fe1—O2^viii^	−152.73 (5)
Fe1—O8—Na7—O4^iv^	−168.78 (8)	Na7^i^—O9—Fe1—O2	27.27 (5)
Fe1—O8—Na7—O5^v^	−54.28 (7)	Na7^i^—O9—Fe1—O8	116.93 (5)
Fe1—O8—Na7—O6	49.67 (6)	Na7^i^—O9—Fe1—O8^viii^	−63.07 (5)
Fe1—O8—Na7—O4^vi^	−143.24 (7)		

Symmetry codes: (i) *x*, −*y*+3/2, *z*−1/2; (ii) −*x*+1, *y*+1/2, −*z*+3/2; (iii) −*x*+2, *y*+1/2, −*z*+3/2; (iv) *x*, −*y*+3/2, *z*+1/2; (v) −*x*+2, *y*−1/2, −*z*+3/2; (vi) −*x*+1, *y*−1/2, −*z*+3/2; (vii) −*x*+1, −*y*+1, −*z*+2; (viii) −*x*+1, −*y*+1, −*z*+1.

### Hydrogen-bond geometry (Å, °)

**Table:**

*D*—H···*A*	*D*—H	H···*A*	*D*···*A*	*D*—H···*A*
O8—H8A···O5^ix^	0.78 (3)	1.97 (3)	2.7513 (15)	172 (2)
O8—H8B···O6^vi^	0.73 (3)	2.00 (3)	2.7116 (15)	165 (2)
O9—H9A···O6^x^	0.74 (3)	2.16 (3)	2.8741 (14)	162 (2)
O9—H9B···O5^ix^	0.70 (3)	2.32 (3)	2.9379 (15)	150 (3)

Symmetry codes: (ix) *x*−1, *y*, *z*; (vi) −*x*+1, *y*−1/2, −*z*+3/2; (x) *x*−1, −*y*+3/2, *z*−1/2.
